# Sex Hormone–Dependent Lipid Mediator Formation in Male and Female Mice During Peritonitis

**DOI:** 10.3389/fphar.2021.818544

**Published:** 2022-01-03

**Authors:** Fabiana Troisi, Simona Pace, Paul M. Jordan, Katharina P. L. Meyer, Rossella Bilancia, Armando Ialenti, Francesca Borrelli, Antonietta Rossi, Lidia Sautebin, Charles N. Serhan, Oliver Werz

**Affiliations:** ^1^ Department of Pharmaceutical/Medicinal Chemistry, Institute of Pharmacy, Friedrich-Schiller-University Jena, Jena, Germany; ^2^ Department of Pharmacy, School of Medicine and Surgery, University of Naples Federico II, Naples, Italy; ^3^ Center for Experimental Therapeutics and Reperfusion Injury, Department of Anesthesia, Perioperative and Pain Medicine, Brigham and Women’s Hospital and Harvard Medical School, Boston, MA, United States of America

**Keywords:** sex differences, lipoxygenase, lipid mediator, specialized pro-resolving mediators, peritonitis, inflammation

## Abstract

**Introduction:** Sex differences in inflammation are obvious and contribute to divergences in the incidence and severity of inflammation-related diseases that frequently preponderate in women. Lipid mediators (LMs), mainly produced by lipoxygenase (LOX) and cyclooxygenase (COX) pathways from polyunsaturated fatty acids (PUFAs), regulate all stages of inflammation. Experimental and clinical studies revealed sex divergences for selected LM pathways without covering the entire LM spectrum, and only few studies have addressed the respective role of sex hormones. Here, we performed the comprehensive LM profile analysis with inflammatory peritoneal exudates and plasma from male and female mice in zymosan-induced peritonitis to identify the potential sex differences in LM biosynthesis during the inflammatory response. We also addressed the impact of sex hormones by employing gonadectomy.

**Methods:** Adult male and female CD1 mice received intraperitoneal injection of zymosan to induce peritonitis, a well-established experimental model of acute, self-resolving inflammation. Mice were gonadectomized 5 weeks prior to peritonitis induction. Peritoneal exudates and plasma were taken at 4 (peak of inflammation) and 24 h (onset of resolution) post zymosan and subjected to UPLC–MS-MS–based LM signature profiling; exudates were analyzed for LM biosynthetic proteins by Western blot; and plasma was analyzed for cytokines by ELISA.

**Results:** Pro-inflammatory COX and 5-LOX products predominated in the peritoneum of males at 4 and 24 h post-zymosan, respectively, with slightly higher 12/15-LOX products in males after 24 h. Amounts of COX-2, 5-LOX/FLAP, and 15-LOX-1 were similar in exudates of males and females. In plasma of males, only moderate elevation of these LMs was apparent. At 4 h post-zymosan, gonadectomy strongly elevated 12/15-LOX products in the exudates of males, while in females, free PUFA and LOX products were rather impaired. In plasma, gonadectomy impaired most LMs in both sexes at 4 h with rather up-regulatory effects at 24 h. Finally, elevated 15-LOX-1 protein was evident in exudates of males at 24 h which was impaired by orchiectomy without the striking impact of gonadectomy on other enzymes in both sexes.

**Conclusions:** Our results reveal obvious sex differences and roles of sex hormones in LM biosynthetic networks in acute self-resolving inflammation in mice, with several preponderances in males that appear under the control of androgens.

## Introduction

Acute inflammation upon tissue damage or invasion of pathogens is an orchestrated response of the immune defense system, regulated by a variety of signaling molecules including lipid mediators (LMs) (Medzhitov 2010; Serhan 2014; Calder P. C. 2020). LMs are polyunsaturated fatty acid (PUFA)-derived metabolites that encompass pro-inflammatory eicosanoids such as prostaglandins (PGs), thromboxanes (TXs), and leukotrienes (LTs) as well as inflammation-resolving specialized pro-resolving mediators (SPMs, i.e., lipoxins, resolvins, maresins, and protectins) that critically regulate the inflammatory response in a temporal manner (Funk 2001; Serhan 2014; Calder P. C. 2020). While PGs/TXs and LTs are rapidly produced at the onset of inflammation where they initialize and maintain the inflammatory process, the formation of SPMs is delayed and is crucial for the termination of inflammation and for the return to homeostasis (Serhan and Savill 2005; Serhan 2014; Serhan and Levy 2018). Imbalances of these pro-inflammatory and inflammation-resolving LMs may result in excessive and persistent inflammation where PG/TX and LT dominate and SPM levels are low (Tabas and Glass 2013; Chiang and Serhan 2020). In fact, chronic inflammatory diseases like asthma and arthritis, cardiovascular diseases, Alzheimer’s disease, type 2 diabetes, and cancer are afflicted with increased ratios of pro-inflammatory eicosanoids vs SPMs (Serhan and Levy 2018; Dalli and Serhan 2019; Chiang and Serhan 2020).

**FIGURE 1:**
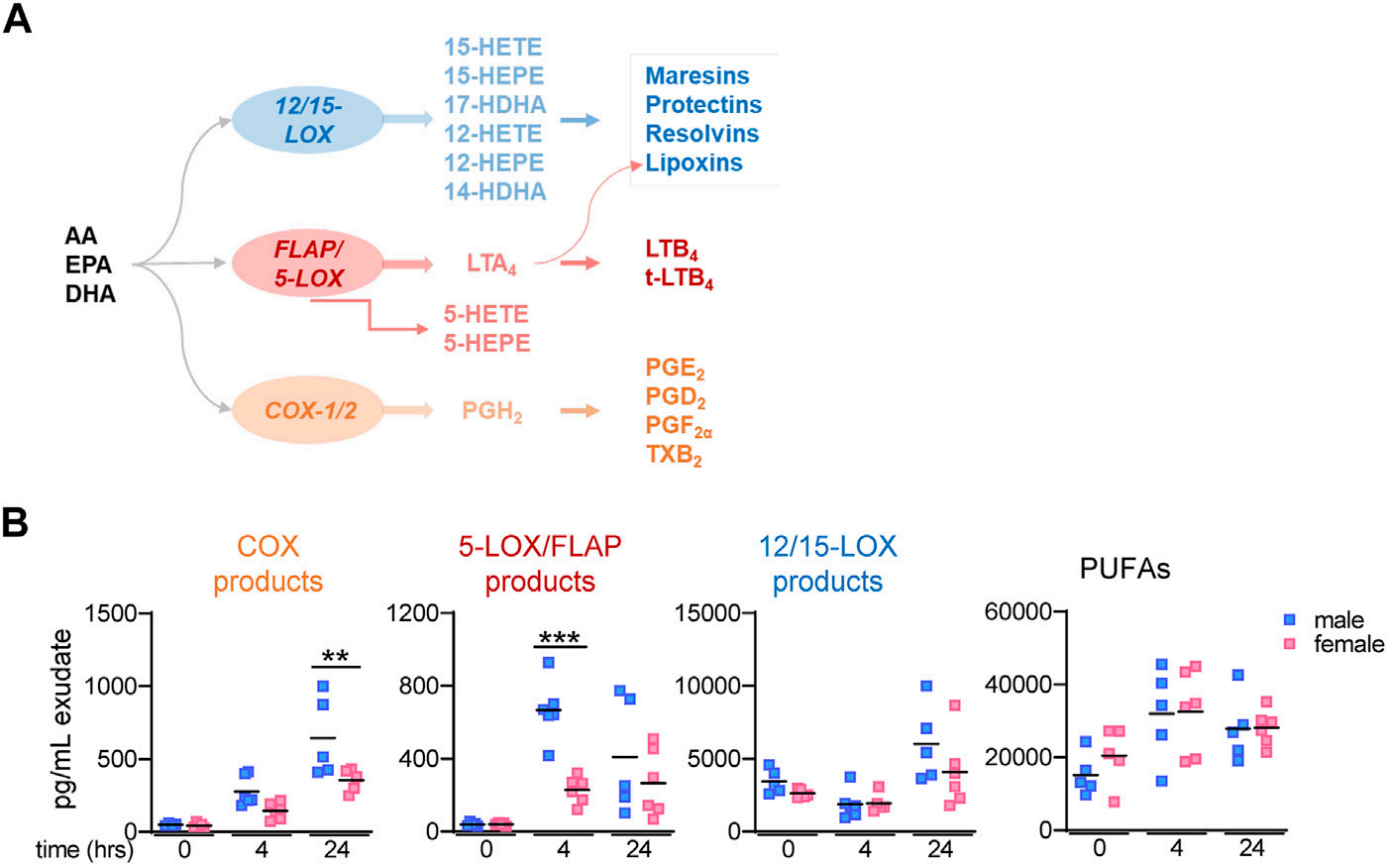
Lipid mediator biosynthetic pathway and lipid mediator levels in mouse peritoneal exudates. **(A)** Scheme of LM biosynthetic pathways yielding 12/15-LOX–, 5-LOX/FLAP–, and COX-1/2–derived products and respective enzymes involved. **(B)** Peritoneal lavages (exudates) from male and female mice were taken at different time points post-zymosan/vehicle i. p. injection. After solid phase extraction, LMs were analyzed by UPLC–MS-MS; time = 0 h: peritoneal lavages immediately taken from mice after treatment with vehicle (no zymosan); t = 4 h and t = 24 h: peritoneal lavages (exudates) post-zymosan. Amounts of relevant LM groups related to respective biosynthetic enzymes: COX products (sum of PGE_2_, PGD_2_, PGF_2α_, and TXB_2_), 5-LOX products (sum of LTB_4_, t-LTB_4_, 5-HETE, and 5-HEPE), 12/15-LOX products (sum of 12-HETE, 12-HEPE, 14-HDHA, 15-HETE, 15-HEPE, and 17-HDHA), and PUFA (sum of AA, EPA, and DHA). Results are shown in pg/mL exudate in scatter dot plots of individual data points and the mean in black lines; n = 5–6; ***p < 0.001 male vs. female; two-way ANOVA with Šídák’s multiple comparison test.

The biosynthesis of LMs is organized in a complex network where multiple enzymes are arranged in connected cascades that act in single cells or *via* transcellular metabolism (Capra et al., 2015; Christie and Harwood 2020). The release of free PUFA from phospholipids by phospholipase A_2_ is a critical step in providing sufficient amounts of arachidonic acid (AA), eicosapentaenoic acid (EPA), and docosahexaenoic acid (DHA) as LM substrates (Astudillo et al., 2019; Murakami et al., 2017). Conversion of AA *via* the cyclooxygenase (COX) pathway yields mainly pro-inflammatory prostanoids such as PGE_2_, PGD_2_, PGF_2_α, and TXA_2_ (Smith et al., 2011), while the AA metabolism *via* the 5-LOX pathway, aided by 5-LOX-activating protein (FLAP), leads to pro-inflammatory LTB_4_ and cysteinyl-containing LT (Radmark et al., 2015) (Figure 1A). In SPM formation, PUFAs are first oxygenated by 12- or 15-LOX to generate 12- or 15-hydroxyeicosatetraenoic acid (12- or 15-HETE) from AA, 12- or 15-hydroxyeicosapentaenoic acid (12- or 15-HEPE) from EPA, and 14- and 17-hydroxydocosahexaenoic acid (14- or 17-HDHA) from DHA, respectively (Chiang and Serhan 2020) (Figure 1A). In a second step, these monohydroxylated products are converted by 12/15-LOXs to generate the di-hydroxylated protectins and maresins, while 5-LOX or 15-LOX accomplish the formation of tri-hydroxylated lipoxins and resolvins *via* hydroperoxidation, epoxide formation, and epoxide hydrolysis; reduction of these hydroperoxides yields di-hydroxylated resolvins (Chiang and Serhan 2020) (Figure 1A).

Sex differences in immune responses are obvious and contribute to divergences in the incidence and severity of inflammation-related disorders, especially autoimmune diseases (Klein and Flanagan 2016; Rosen et al., 2017). Notably, a variety of inflammatory diseases related to elevated PG and LT levels are sex-biased and preponderate in women, for example, rheumatoid arthritis and asthma (Pace et al., 2017c; Pace and Werz 2020). The side-by-side analysis of LT formation in neutrophils, monocytes, and macrophages from male and female human beings and/or rodent subjects revealed superior LT levels in samples from females (Pergola et al., 2008; Pergola et al., 2011; Rossi et al., 2014; Pace et al., 2017a). Androgens are causative for these sex divergences, as they suppressed LT formation by preventing the intracellular assembly of the LT-biosynthetic 5-LOX/FLAP complex (Pergola et al., 2008; Pace et al., 2017a), involving extracellular signal–regulated protein kinase (ERK)-1/2 (Pergola et al., 2008) and/or phospholipase (PL)D (Pergola et al., 2011) signaling routes. In an asthma model, LT levels increased only in lungs of female but not in those of male mice during allergen sensitization along with superior airway hyperreactivity and pulmonary inflammation (Rossi et al., 2019). Androgen administration to sensitized female mice abrogated this sex bias in LT-mediated asthma (Cerqua et al., 2020). Also sex differences in the PG biosynthesis were reported (for review, see Pace et al. (2017c)), where PG levels often dominate in males, even though opposite findings were apparent, depending on the type of organ or tissue and the experimental settings, for example, the time point of analysis and age of the subjects with consequences for the hormonal status (Pace et al., 2017a; Pace et al., 2017b). Finally, sex differences in the SPM biosynthesis were evident upon the analysis of plasma from healthy or diseased humans and from experimental studies with humans or rodents. Obviously, EPA and DHA are more efficiently biosynthesized in females, and females have higher plasma levels of DHA than males (Calder PC. 2020). However, there is currently no consistent sex bias in the levels of SPM and their precursors, which in some studies dominate in males (English et al., 2017; Barden et al., 2018; Pullen et al., 2020), while in other studies, they preponderate in female subjects (Barden et al., 2015; Rathod et al., 2017; Halade et al., 2020; Trotta et al., 2021; Yaeger et al., 2021). Moreover, the influence of sex hormones on the SPM biosynthesis is rather elusive.

There is obviously no consisting pattern of sex differences that unequivocally proves preponderances of all different LMs in males or females. With some few exceptions (English et al., 2017; Rathod et al., 2017; Yaeger et al., 2021), most studies addressing sex differences and/or the impact of sex hormones related to the LM biosynthesis focused on single selected pathways, namely, either 5-LOX/LTs or COX/prostanoids, or SPM, and neglected the others. Here, we aimed at obtaining comprehensive LM signature profiles including pro-inflammatory LT and PG/TX and inflammation-resolving SPM in inflammatory exudates and plasma from male and female mice at the peak (4 h) and the resolution (24 h) of acute inflammation employing zymosan-induced peritonitis as an appropriate experimental model (Cash et al., 2009). Moreover, using this model, we aimed at revealing the impact of sex hormones on the LM biosynthesis in acute inflammation by employing gonadectomy of male and female mice in order to abrogate endogenous sex hormone production (Scotland et al., 2011; Rossi et al., 2014).

## Materials and Methods

### Animals

Adult (5–6 weeks) male and female CD1 mice (Charles River, Calco, Italy) were housed at the animal care facility of the Department of Pharmacy of the University of Naples “Federico II” and kept under controlled environment (i.e., temperature 21 ± 2°C and humidity 60 ± 10%) with free access to normal chow and water. Experiments were conducted after 4 days of acclimation of the mice during the light phase of a 12-h light/dark schedule. The experimental procedures were approved by the Italian Ministry and carried out in accordance with the EU Directive 2010/63/EU and the Italian DL 26/2014 for animal experiments, and in compliance with the ARRIVE guidelines and Basel declaration including the 3R concept.

### Surgical Removal of Gonads

In order to investigate the impact of sex hormones, male and female mice underwent gonadectomy. Orchidectomy was performed as previously described (Rossi et al., 2014). In brief, male mice were anesthetized using xylazine (10 mg/kg) and ketamine (100 mg/kg), and subsequently immobilized, shaved, and disinfected. Testes were removed through a perineal raphe access. Then, the scrotum was closed by a single stitch. For the ovariectomies, female mice were immobilized with a ventral decubitus after anesthesia, and ovaries were removed by applying two cuts on the lateral side of the rachis (left and right); then, a suture with a single stitch was applied. For both groups of mice with orchidectomy and ovariectomy, respective control groups (male and female) that were “sham-operated” without the removal of the gonads were used. Animals were allowed to recover for 5 weeks after the surgery which is the time necessary for the turnover of the sexual hormone levels in the body.

### Zymosan-Induced Peritonitis in Mice

Peritonitis was induced in male and female “sham-operated” and gonadectomized mice (five to six per group), respectively, 5 weeks after surgery. The protocol utilized for the induction of peritonitis followed the procedures and conditions that were described by us before (Rossi et al., 2014; Pace et al., 2017a; Pace et al., 2017b). Briefly, after intraperitoneal (i.p.) injection of 1 mg zymosan as suspension in 0.5 ml of saline per mouse, the animals were euthanized in a saturated atmosphere with CO_2_ at selected time points (4 and 24 h post-zymosan), and blood and peritoneal exudates were collected. A control group “time zero” (no zymosan) for sham-operated and gonadectomized mice was included. Peritoneal exudates were obtained by washing the cavity with 3 ml of sterile filtered cold PBS. These peritoneal exudates were then centrifuged at 20,000 × g for 20 min in order to remove cells. Samples were immediately frozen at −80°C. Blood (0.7–0.9 ml) was collected by intracardiac puncture (22-gauge needle) using citrate as an anticoagulant (3.8% (w/v)), immediately after killing of mice with CO_2_, and centrifuged at 800 × g at 4 °C for 10 min in order to obtain plasma. Cell-free exudates and plasma were used for the measurements of LMs, Western blot analysis, and cytokine analysis.

### Cytokine Analysis

ELISA kits for the detection of murine IL-1β, IL-6, and TNF-α in peritoneal exudates (R&D systems, Wiesbaden-Nordenstadt, Germany) were used according to the instructions of the manufacturers.

### Sample Preparation and Solid Phase Extraction of Lipid Mediators

Peritoneal exudates (1 ml) and plasma (0.15 or 0.2 ml) were diluted in ice-cold methanol (ratio 1:3, v/v). Deuterium-labeled internal standards were added to each sample, as follows: d8-5S-HETE, d4-PGE_2_, d4-LTB_4_, d5-LXA_4_, d5-RvD2, and d8-AA (500 pg, each). Samples were kept in methanol at −20°C for 60 min to allow protein precipitation and then centrifuged (1,200 × g, 4 °C, 10 min). Samples were diluted with 8 ml of acidified water (pH 3.5) immediately before solid phase extraction. The solid phase C18 cartridges (SepPak Vac C18 6cc, Waters, Milford, MA, United States) were equilibrated with 6 ml of methanol before the addition of 2 ml of water. Acidified samples were loaded onto the conditioned C18 cartridges and washed with 6 ml of water and 6 ml of *n*-hexane. LMs were eluted with 6 ml of methyl formate. Subsequently, eluted samples (6 ml) were evaporated until dryness using an evaporation system (TurboVap LV, Biotage, Uppsala, Sweden) with nitrogen (5–10 psi). Samples were then resuspended in 100 µL methanol/water (1:1, v/v) and centrifuged twice (15,000 × g, 5 min, 4 °C) for UPLC–MS-MS automated injections.

### Analysis of Lipid Mediators by UPLC–MS-MS

LMs were analyzed with an Acquity™ UPLC system (Waters, Milford, MA, United States) and a QTRAP 5500 mass spectrometer (ABSciex, Darmstadt, Germany) equipped with a Turbo V™ Source and electrospray ionization. LMs were separated using an ACQUITY UPLC^®^ BEH C18 column (1.7 µm, 2.1 × 100 mm; Waters, Eschborn, Germany) at 50°C with a flow rate of 0.3 ml/min and a mobile phase consisting of methanol–water–acetic acid of 42:58:0.01 (v/v/v) that was ramped to 86:14:0.01 (v/v/v) over 12.5 min and then to 98:2:0.01 (v/v/v) for 3 min (Werner et al., 2019). QTRAP 5500 was operated in the negative ionization mode using scheduled multiple reaction monitoring (MRM) coupled with information-dependent acquisition. The scheduled MRM window was 60 s, optimized LM parameters were adopted (Werner et al., 2019), and the curtain gas pressure was set to 35 psi. The retention time and at least six diagnostic ions for each LM were confirmed by means of an external standard (Cayman Chemical/Biomol GmbH, Hamburg, Germany). Quantification was achieved by calibration curves for each LM. Linear calibration curves were obtained for each LM and gave *r* ^2^ values of 0.998 or higher. Additionally, the limit of detection for each targeted LM was determined (Werner et al., 2019).

### Western Blot Analysis

Exudates corresponding to equal amounts of proteins were separated by electrophoresis on SDS–polyacrylamide gels (10% for 5-LOX, p12-LOX, 15-LOX-1, COX-2, and *β*-actin; 16% for FLAP) and blotted onto nitrocellulose membranes (Hybond ECL, GE Healthcare, Freiburg, Germany). Ponceau in 5% (v/v) acetic acid was used as a stain to confirm the correct loading and protein transfer to the membrane. After blocking with 0.1% TBS/Tween containing 5% BSA (1 h, room temperature), the membranes were washed and incubated overnight at 4 °C with primary antibodies as follows: monoclonal mouse anti–5-LOX-1:1,000 (Clone 33/5-Lipoxygenase, BD Biosciences, San Jose, CA, United States); polyclonal rabbit anti–15-LOX-1, 1:200 (Abcam, ab80221); COX-2 rabbit, polyclonal 1:1,000 (Cat#4842S, Cell Signaling, Danvers, MA, United States); FLAP rabbit, polyclonal 1:1,000 (Cat#ab85227, Abcam, Cambridge, UK); platelet-type 12-LOX mouse monoclonal, 1:300 (Cat#sc-365194, Santa Cruz Biotechnology, Heidelberg, Germany); and *β*-actin rabbit, monoclonal 1:1,000 (Cat#3700, Cell Signaling). After incubation, membranes were washed with 0.1% TBS-Tween (3 × 10 s) and were incubated for 1.5 h at room temperature with fluorescence-labeled secondary antibodies IRDye 800 CW (dilution 1:10.000) and IRDye 680 LT (dilution 1:80.000) for 1 h, at RT. After washing and overnight drying, immune-reactive bands were visualized by an Odyssey infrared imager (LI-COR Biosciences).

### Statistical Analysis

Results are expressed as mean ± S.E.M. of the mean of *n* observations, where *n* represents the number of animals or number of samples analyzed. Results were analyzed by 2-tailed Student’s *t* test, one-way ANOVA followed by Tukey *post hoc* tests, or two-way ANOVA followed by Šídák’s multiple comparison test. A *p*-value less than 0.05 was considered significant. Outliers were identified by Grubbs’outlier test.

## Results

### Sex Differences in the Lipid Mediator Formation in Zymosan-Challenged Peritoneum of Mice

In order to investigate if LM signature profiles produced during acute inflammation differ in mammals between sexes, we employed the well-established and suitable model of zymosan-induced peritonitis in male and female mice (Cash et al., 2009; Rossi et al., 2014). Targeted LM metabololipidomics using UPLC–MS-MS of peritoneal exudates were obtained from mice 4 h after zymosan injection, which is the temporal peak of inflammatory reactions (Rossi et al., 2014), and 24 h post-zymosan, which corresponds to the onset of inflammation resolution (Serhan, 2014). In addition, exudates from mice where vehicle was injected i.p. instead of zymosan and sacrificed right thereafter (designated as t = 0) were used as samples of unstimulated “healthy” controls. For the sake of simplicity in presentation, we grouped LMs into COX products (sum of PGE_2_, PGD_2_, PGF_2α_, and TXB_2_), 5-LOX products (sum of LTB_4_, t-LTB_4_, 5-HETE, and 5-HEPE), 12/15-LOX products (sum of 12-HETE, 12-HEPE, 14-HDHA, 15-HETE, 15-HEPE, and 17-HDHA), and PUFAs (sum of AA, EPA, and DHA). In vehicle-treated “healthy” mice (t = 0), COX and 5-LOX products in the exudates were low, while substantial amounts of 12/15-LOX products and PUFAs were detectable, without obvious sex differences for any LM group (Figure 1B). At 4 and 24 h post-zymosan, COX and 5-LOX products were strongly elevated with a tendency of higher levels in males vs females, being significant for 5-LOX products at 4 h and for COX products at 24 h (Figure 1B). In contrast, the amounts of 12/15-LOX products at 4 h were not changed vs vehicle-treated mice but elevated at 24 h after zymosan with a higher tendency in males. Moderate elevations without differences between sexes were found for PUFAs (Figure 1B).

More detailed analysis of single LM members within the COX/LOX groups at t = 0, 4, and 24 h is shown in Table 1. Among COX products, PGE_2_ levels were somewhat elevated in males vs females at all three time points, but PGF_2_α and TXB_2_ clearly dominated in exudates of males, especially at 4 h. Similarly, all 5-LOX products were higher in exudates of males after 4 and 24 h, especially the major products, namely, LTB_4_ and 5-HETE. In contrast, 12/15-LOX-derived 14-HDHA, 17-HDHA, and 15-HEPE were higher in female mice but only at 4 h, even though RvD5 was lower. At t = 0 and at 24 h, these 12/15-LOX products were hardly different between the sexes or somewhat lower in females (Table 1). Taken together, sex differences in LM levels in exudates during acute peritonitis are obvious not only for pro-inflammatory COX and 5-LOX products that were elevated in males at 4 and 24 h but also for 12/15-LOX products elevated in males at 24 h, without accompanying the differences in free PUFA levels.

**TABLE 1:** Lipid mediator profiling in peritoneal exudates of male and female mice. Amounts of LM in peritoneal exudates from male and female mice taken at different time points post-zymosan/vehicle i.p. injection (see legends Figure 1). Data are given in pg/mL exudate expressed as mean ± SEM of n = 5 to 6 animals per group and as heatmap. Fold-change of female mean values compared to male mean values. *p < 0.05, **p < 0.01 male 0 h vs. Four or 24 h; #*p* < 0.05, ##*p* < 0.01 female 0 h vs. Four or 24 h; unpaired Student’s *t* test.

No	**0 h**			**4 h**			**24 h**		
	**Male**	**Female**	**Fold**	**Male**	**Female**	**Fold**	**Male**	**Female**	**Fold**
**COX products**									
PGE_2_	13 ± 2.3	12 ± 1.5	0.9	41 ** ± 6.9	34^##^ ± 4.2	0.8	80 * ± 18	54^##^ ± 3.7	0.7
PGD_2_	5.6 ± 1.1	3.2 ± 0.5	0.6	18 ** ± 2.6	10^##^ ± 1.4	0.6	23 * ± 5.1	16^##^ ± 1.2	0.7
PGF_2_	7.7 ± 2.9	4.3 ± 0.6	0.6	106 ** ±18	41^#^ ± 9.4	0.4	230 ** ± 23	115^##^ ± 10	0.5
TXB_2_	38 ± 9.1	24 ± 6.6	0.6	113 ** ±18	60^#^ ± 8.9	0.5	314 * ± 81	189^##^± 20	0.6
**5-LOX products**									
LTB_4_	n.d	n.d	-	65 ** ± 8.5	17^##^± 2.4	0.3	32 ± 14	21^#^ ± 7.5	0.7
t-LTB_4_	21 ± 4.1	15 ± 1.4	0.7	15 ± 1.7	6.7^##^ ± 1.0	0.4	17 ± 4.0	15 ± 3.9	0.9
5-HEPE	3.5 ± 0.6	5.7 ± 1.7	1.6	48 ** ± 6.5	26^##^ ± 4.9	0.5	26 ± 11	15^#^ ± 2.7	0.6
5-HETE	16 ± 2.1	20 ± 2.9	1.3	538** ±56	179^##^ ± 23	0.3	334 * ± 114	216^#^ ± 62	0.6
**12/15-LOX products**									
14-HDHA	339 ± 49	286 ± 23	0.8	266 ± 41	534 ± 164	2.0	767 ± 180	669 ± 165	0.9
12-HEPE	1,393 ± 144	1,000 ± 67	0.7	534** ± 110	567^##^ ± 78	1.1	2,186 ± 408	1,536 ± 408	0.7
12-HETE	1,327 ± 137	952 ± 64	0.7	509** ± 105	540^##^ ± 74	1.1	2,082 ± 389	1,001 ± 162	0.5
17-HDHA	84 ± 22	91 ± 12	1.1	101 ± 13	186 ± 46	1.8	317 ± 97	278^#^ ± 66	0.9
15-HEPE	43 ± 3.8	53 ± 6.6	1.2	35 ± 3.1	68 ± 8.3	2.0	90 * ± 13	65 ± 10	0.7
15-HETE	239 ± 57	235 ± 20	1.0	249 ± 24	268 ± 42	1.1	561 * ± 105	283 ± 50	0.5
PDX	<3	<3	-	n.d	n.d	-	n.d	n.d	
PD1	<3	<3	-	n.d	n.d	-	3.8 ± 0.8	5.9 ± 1.7	1.6
RvD5	4.5 ± 0.5	n.d	-	12 ± 2.1	4.0^##^ ± 0.7	0.3	4.5 ± 2.0	3.5^#^ ± 1.3	0.8
LXA_4_	<3	<3	-	n.d	n.d	-	<3	<3	-
**PUFAs**									
AA	10,790 ± 1,496	14,536 ± 2,422	1.3	18,086 * ± 2,614	17,695 ± 2,126	1.0	19,187 * ± 2,092	18,697 ± 1,716	1.0
EPA	834 ± 106	847 ± 162	1.0	1,950* ± 417	2,135^#^ ± 474	1.1	1,682 ± 411	1,471^#^ ± 183	0.9
DHA	3,499 ± 968	5,111 ± 1,222	1.5	11,935 * ± 2,679	12,755^#^± 2,120	1.1	7,022 ± 1,488	7,941 ± 442	1.1

#### Gonadectomy-Mediated Sex Hormone Depletion Differentially Affects Lipid Mediator Abundance in Male and Female Mice.

**FIGURE 2:**
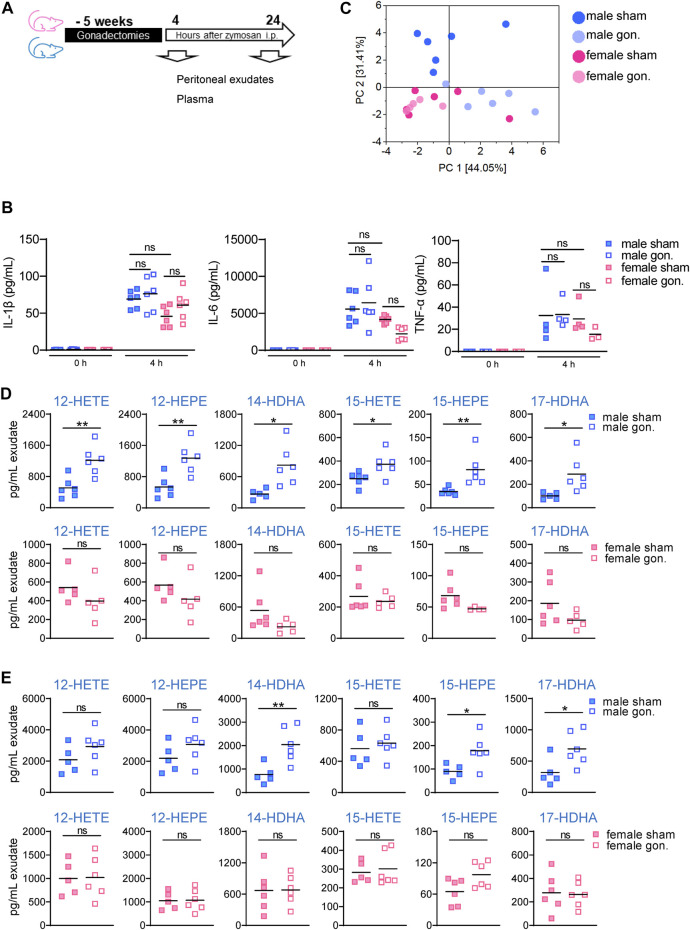
Effect of hormone depletion on the LM content in peritoneal exudates from male and female mice upon peritonitis induction. **(A)** Experimental setup and time line. **(B)** Amounts of pro-inflammatory cytokines IL-1β, IL-6, and TNF-α in peritoneal exudates of gonadectomized and non-gonadectomized (sham-operated) male and female mice after treatment with vehicle (no zymosan = 0 h) and 4 h after zymosan injection. Results are shown in pg/mL exudate in scatter dot plots as individual data points and means as black line; n = 4–6; ordinary one-way ANOVA with Tukey’s multiple comparison test. **(C)** Principal component analysis of LM profiles in the exudates from gonadectomized and non-gonadectomized (sham-operated) male and female mice, 4 h after zymosan injection (corresponding raw data are shown in Table 2). **(D,E)** Amounts of 12/15-LOX products in peritoneal exudates of gonadectomized and non-gonadectomized (sham-operated) male and female mice 4 h **(D)** and 24 h **(E)** after zymosan injection. Results are shown in pg/mL exudate in scatter dot plots as individual data points and the means as black lines; n = 5–6; *p < 0.05, **p < 0.01 non-gonadectomized vs. gonadectomized; unpaired Student’s *t* test; ns = not significant.

Since sex hormones are known to affect 5-LOX product levels *in vitro* and *in vivo*, where androgens such as 5α-DHT act as endogenous suppressors in neutrophils, monocytes, and macrophages (Pace et al., 2017a; Pergola et al., 2008; Pergola, et al., 2011), and progesterone down-regulates LTs in monocytes (Pergola et al., 2015), we studied how ablation of sex hormone production in mice would affect LM levels during subsequently induced peritonitis. Male and female mice were gonadectomized or sham-operated and allowed to recover for 5 weeks after surgery, which is the time necessary for the turnover of the sex hormone levels (Scotland et al., 2011; Rossi et al., 2014). Then, zymosan was injected, and animals were sacrificed after 4 and/or 24 h for the analysis of cytokines and LMs in peritoneal exudates and systemically in plasma (Figure 2A).

The levels of the pro-inflammatory cytokines IL-1β, TNFα, and IL-6 in the exudates 4 h post-zymosan were not significantly different between the sexes of sham-operated mice, although a tendency for elevated levels of IL-1β and IL-6 in exudates of males vs females was observed (Figure 2B). Also, gonadectomy did not significantly alter peritoneal cytokine levels in either sex, but a trend toward lower levels for TNFα and IL-6 was seen in female but not in male mice (Figure 2B). At 4 h post-zymosan, pro-inflammatory 5-LOX products were lower in the exudates after gonadectomy of females but also of males, while the amounts of COX products in parallel were somewhat increased, except PGF_2_α in males (Table 2). In contrast, significant and striking elevation of the 12/15-LOX products 17-HDHA, 14-HDHA, 15-HETE, 15-HEPE, 12-HETE, and 12-HEPE was evident after orchiectomy in exudates of male mice but rather impaired in those of female animals after ovariectomy (Table 2). Gonadectomy caused lower free PUFA levels in exudates of female mice with only very small effects in males (Table 2). We found marked differences in the LM profiles between the various groups of mice by employing the unbiased principal component analysis (PCA). In detail, LM metabolomes from male sham-operated mice clustered in a different manner from those derived from other mice, implying an obvious impact of sex hormones, seemingly androgens (Figure 2C).

**TABLE 2:** Effect of hormone depletion on lipid mediator profiles in peritoneal exudates from male and female mice at 4 h post-zymosan injection. Amounts of LM in peritoneal exudates from male and female mice 4 h post zymosan/vehicle i.p. injection that underwent gonadectomy or sham-operation (see legend Figure 2). Data are given in pg/mL exudate expressed as mean ± SEM of n = 5 to 6 animals per group and as heatmap. Fold-change of gonadectomy mean values compared to sham mean values of male and female mice.

No	**4 h**					
	**Male sham**	**Male gon**	Fold	**Female sham**	**Female gon**	Fold
**COX products**						
PGE_2_	41 ± 6.9	71 ± 8.9	1.7	34 ± 4.2	43 ± 3.2	1.3
PGD_2_	18 ± 2.6	22 ± 3.1	1.2	10 ± 1.4	12 ± 1.2	1.2
PGF_2_	106 ± 18	61 ± 12	0.6	41 ± 9.4	53 ± 5.8	1.3
TXB_2_	113 ± 18	139 ± 19	1.2	60 ± 8.9	81 ± 8.2	1.4
**5-LOX products**						
LTB_4_	65 ± 8.5	25 ± 3.2	0.4	17 ± 2.4	11 ± 2.4	0.7
t-LTB_4_	15 ± 1.7	9.0 ± 0.9	0.6	6.7 ± 1.0	5.1 ± 0.9	0.8
5-HEPE	48 ± 6.5	20 ± 4.4	0.4	26 ± 4.9	6.6 ± 1.1	0.3
5-HETE	538 ± 56	244 ± 51	0.5	179 ± 23	69 ± 11	0.4
**12/15-LOX products**						
14-HDHA	266 ± 41	821 ± 159	3.1	534 ± 164	220 ± 51	0.4
12-HEPE	534 ± 110	1,275 ± 161	2.4	567 ± 78	417 ± 96	0.7
12-HETE	509 ± 105	1,214 ± 153	2.4	540 ± 74	397 ± 91	0.7
17-HDHA	101 ± 13	287 ± 62	2.8	186 ± 46	96 ± 19	0.5
15-HEPE	35 ± 3.1	82 ± 14	2.3	68 ± 8.3	47 ± 1.2	0.7
15-HETE	249 ± 24	372 ± 43	1.5	268 ± 42	236 ± 19	0.9
RvD5	12 ± 2.2	4.6 ± 1.3	0.4	4.0 ± 0.7	<3	
**PUFAs**						
AA	18,086 ± 2,614	16,158 ± 2,011	0.9	17,695 ± 2,126	8,763 ± 620	0.5
EPA	1,950 ± 417	1,341 ± 206	0.7	2,135 ± 474	943 ± 186	0.4
DHA	11,935 ± 2,679	9,638 ± 1,630	0.8	12,755 ± 2,120	3,874 ± 524	0.3

The increased levels of 12/15-LOX products in exudates of male mice after orchiectomy were essentially maintained also at 24 h post-zymosan, where again in female animals such elevations due to ovariectomy were absent, except a tendency for 15-HEPE (Table 3; Figure 2D). Again, ovariectomy caused lower 5-LOX products in exudates of females at 24 h after zymosan, while in contrast to 4 h, 5-LOX products were slightly elevated in male exudates due to orchiectomy (Table 3). After 24 h, COX products in exudates were rather decreased due to gonadectomy, particularly in female mice. Note that PUFA levels were up-regulated in male exudates after orchiectomy and to some extent, at least for EPA, also in female animals due to ovariectomy (Table 3). To sum up, gonadectomy strongly up-regulates 12/15-LOX products in peritoneal exudates of male mice 4 and 24 h post-zymosan, along with elevated PUFAs and 5-LOX products after 24 h, but not so in female mice, where free PUFA (after 4 h) and 5-LOX products (4 and 24 h) are lowered as a consequence of ovariectomy. Therefore, in this experimental model, androgens may repress PUFA release and 12/15-LOX product formation in males at 4 and 24 h, while progesterone and/or estrogens are rather stimulatory in this respect.

**TABLE 3:** Effect of hormone depletion on lipid mediator profiles in peritoneal exudates from male and female mice at 24 h post-zymosan injection. Amounts of LM in peritoneal exudates from male and female mice 24 h post-zymosan/vehicle i.p. injection that underwent gonadectomy or sham-operation (see legend Figure 2). Data are given in pg/mL exudate expressed as mean ± SEM of n = 5 to 6 animals per group and as heatmap. Fold-change of gonadectomy mean values compared to sham mean values of male and female mice.

No	**4 h**					
	**Male sham**	**Male gon**	Fold	**Female sham**	**Female gon**	Fold
**COX products**						
PGE_2_	80 ± 18	75 ± 5.6	0.9	54 ± 3.7	42 ± 6.8	0.8
PGD_2_	23 ± 5.1	29 ± 5.6	1.3	16 ± 1.2	12 ± 2.3	0.7
PGF_2_	230 ± 23	159 ± 11	0.7	115 ± 10	100 ± 16	0.9
TXB_2_	314 ± 81	282 ± 29	0.9	189 ± 20	147 ± 36	0.8
**5-LOX products**						
LTB_4_	32 ± 14	45 ± 9.2	1.4	21 ± 7.5	5.2 ± 0.7	0.2
t-LTB_4_	17 ± 4.0	23 ± 3.2	1.3	15 ± 3.9	6.5 ± 0.7	0.4
5-HEPE	26 ± 11	50 ± 13	1.9	15 ± 2.7	17 ± 3.1	1.2
5-HETE	334 ± 114	459 ± 76	1.4	216 ± 62	120 ± 31	0.6
**12/15-LOX products**						
14-HDHA	767 ± 180	2,041 ± 304	2.7	669 ± 165	680 ± 115	1.0
12-HEPE	2,186 ± 408	3,072 ± 453	1.4	1,052 ± 170	1,072 ± 188	1.0
12-HETE	2,082 ± 389	2,926 ± 431	1.4	1,001 ± 162	1,021 ± 179	1.0
17-HDHA	317 ± 97	696 ± 111	2.2	278 ± 66	264 ± 45	1.0
15-HEPE	90 ± 13	178 ± 27	2.0	65 ± 10	97 ± 10	1.5
15-HETE	561 ± 105	632 ± 78	1.1	283 ± 50	301 ± 38	1.1
PD1	3.8 ± 0.8	12 ± 1.0	3.1	5.9 ± 1.7	6.7 ± 1.2	1.1
RvD5	4.5 ± 2.0	7.3 ± 1.1	1.6	3.5 ± 1.3	<3	
LXA_4_	<3	<3	-	<3	<3	-
**PUFAs**						
AA	19,187 ± 2,092	26,529 ± 929	1.4	18,697 ± 1,716	19,272 ± 2,501	1.0
EPA	1,682 ± 411	3,271 ± 562	1.9	1,471 ± 183	2,377 ± 275	1.6
DHA	7,022 ± 1,488	13,950 ± 1,382	2.0	7,941 ± 442	9,044 ± 703	1.1

**FIGURE 3:**
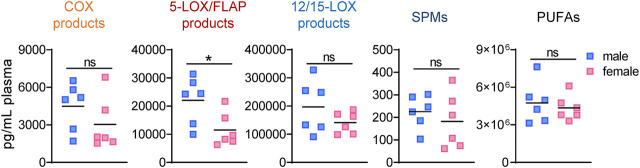
Lipid mediator levels in plasma of male and female mice during peritonitis. Amounts of COX products (sum of PGE_2_, PGD_2_, PGF_2α_, and TXB_2_), 5-LOX products (sum of LTB_4_, t-LTB_4_, 5-HETE, and 5-HEPE), 12/15-LOX products (sum of 12-HETE, 12-HEPE, 14-HDHA, 15-HETE, 15-HEPE, and 17-HDHA), and PUFA (sum of AA, EPA, and DHA) in plasma of male and female mice 4 h after zymosan injection. Results are shown in pg/mL plasma in scatter dot plots as individual data points and the means as black lines; n = 5–6; *p < 0.05, male vs. female; unpaired Student’s *t* test; ns = not significant.

The analysis of LM profiles in plasma of the respective animals showed divergent effects due to gonadectomy, especially in males at 4 h post-zymosan. As shown in Figure 3 and Table 4, the levels of PUFAs, COX, and 12/15-LOX products, and SPMs at 4 h post-zymosan were not significantly different between the sexes (no gonadectomy), while 5-LOX products were higher in plasma from males vs females. At 24 h post-zymosan, COX and 5-LOX product levels were minute, while 12/15-LOX products were still abundant and clearly higher in plasma of male mice; PUFA levels hardly differed between the sexes (Table 5). Orchiectomy caused an overall decrease of COX, 5-LOX, and 12/15-LOX products after 4 h of zymosan in plasma (Table 4). Note that PUFA levels were not impaired, but AA and EPA were rather elevated after gonadectomy. For female animals 4 h post-zymosan, ovariectomy lowered plasma levels of 5-LOX and 12/15-LOX products like in exudates, and strongly suppressed the amounts of COX products (Table 4), which were instead elevated in the corresponding exudates. Like in male mice, plasma PUFA levels in female animals were not altered due to gonadectomy, which is in contrast to the corresponding exudates (Table 4). Interestingly, these main detrimental effects of gonadectomy in male and female mice on LM plasma levels after 4 h vanished after 24 h of zymosan, and a small trend toward higher levels of LOX products and PUFAs became apparent (Table 5). It should be noted that COX products were sparsely present in plasma, regardless of gonadectomy (Table 5). Together, despite elevated PUFA levels in plasma, gonadectomy causes impaired levels of COX, 5-LOX, and 12/15-LOX products in both sexes at 4 h, but not at 24 h.

**TABLE 4:** Effect of hormone depletion on lipid mediator profiles in plasma from male and female mice at 4 h post-zymosan injection. Amounts of LM in plasma from male and female mice 4 h post zymosan/vehicle i.p. injection that underwent gonadectomy or sham-operation. Data of n = 5 to 6 animals per group are shown as mean ± SEM in pg/mL plasma and as heatmap. Fold-change of gonadectomy mean values compared to sham mean values of male and female mice.

No	**4 h**					
	**Male sham**	**Male gon**	Fold	**Female sham**	**Female gon**	Fold
**COX products**						
PGE_2_	2,512 ± 487	790 ± 237	0.3	1,581 ± 494	234 ± 139	0.1
PGD_2_	1,365 ± 262	504 ± 163	0.4	896 ± 292	142 ± 87	0.2
PGF_2_	177 ± 22	58 ± 12	0.3	117 ± 20	14 ± 10	0.1
TXB_2_	434 ± 17	272 ± 31	0.6	439 ± 54	93 ± 46	0.2
**5-LOX products**						
LTB_4_	140 ± 20	39 ± 7.2	0.3	100 ± 34	66 ± 16	0.7
t-LTB_4_	311 ± 51	85 ± 26	0.3	175 ± 55	83 ± 13	0.5
5-HEPE	1,075 ± 87	672 ± 99	0.6	726 ± 84	892 ± 106	1.2
5-HETE	20,508 ± 3,305	7,770 ± 2,085	0.4	10,531 ± 2,388	4,516 ± 927	0.4
**12/15-LOX products**						
14-HDHA	66,682 ± 15,812	37,727 ± 4,506	0.6	53,620 ± 5,559	32,120 ± 4,562	0.6
12-HEPE	18,230 ± 5,189	7,142 ± 1,154	0.4	12,196 ± 2,055	10,382 ± 1890	0.9
12-HETE	82,404 ± 16,431	45,061 ± 3,486	0.5	59,519 ± 6,788	34,559 ± 6,476	0.6
17-HDHA	5,060 ± 535	2,478 ± 339	0.5	3,131 ± 441	1,660 ± 93	0.5
15-HEPE	409 ± 22	276 ± 41	0.7	314 ± 50	387 ± 23	1.2
15-HETE	24,205 ± 2,339	7,698 ± 1,630	0.3	12,563 ± 2,739	5,257 ± 994	0.4
PD1	29 ± 2.6	n.d		22 ± 3.5	n.d	
PDX	58 ± 5.3	27 ± 3.6	0.5	51 ± 13	22 ± 4.1	0.4
MaR1	90 ± 19	n.d	-	80 ± 29	n.d	-
RvD1	289 ± 38	85 ± 22	0.3	248 ± 80	29 ± 16	0.1
RvD4	2,412 ± 399	920 ± 280	0.4	1,278 ± 425	514 ± 154	0.4
RvD5	49 ± 5.9	n.d	-	30 ± 12	n.d	-
**PUFAs**						
AA	2,686,452 ± 344,713	3,494,389 ± 268,824	1.3	2,654,887 ± 265,662	2,630,517 ± 443,140	1.0
EPA	692,395 ± 117,398	966,984 ± 86,084	1.4	736,517 ± 66,330	992,826 ± 158,138	1.3
DHA	1,376,142 ± 250,885	1,214,286 ± 141,497	0.9	959,017 ± 163,038	1,174,683 ± 317,718	1.2

**TABLE 5:** Effect of hormone depletion on lipid mediator profiles in plasma from male and female mice at 24 h post-zymosan injection. Amounts of LM in plasma from male and female mice 24 h post zymosan/vehicle i.p. injection that underwent gonadectomy or sham-operation. Data of n = 5 to 6 animals per group are shown as mean ± SEM in pg/mL plasma and as heatmap. Fold-change of gonadectomy mean values compared to sham mean values of male and female mice.

No	**24 h**					
	**Male sham**	**Male gon**	Fold	**Female sham**	**Female gon**	Fold
**COX products**						
PGE_2_	28 ± 3.0	30 ± 4.5	1.0	n.d	n.d	
PGD_2_	n.d	26 ± 5.4		n.d	n.d	
PGF_2_	n.d	n.d		n.d	n.d	
TXB_2_	100 ± 32	127 ± 32	1.3	n.d	n.d	
**5-LOX products**						
LTB_4_	n.d	n.d	-	n.d	n.d	-
t-LTB_4_	n.d	n.d	-	n.d	n.d	-
5-HEPE	951 ± 95	540 ± 57	0.6	469 ± 70	623 ± 29	1.3
5-HETE	906 ± 91	1,158 ± 114	1.3	827 ± 82	1,076 ± 100	1.3
**12/15-LOX products**						
14-HDHA	16,370 ± 3,713	21,385 ± 5,322	1.3	7,245 ± 1,534	10,127 ± 3,614	1.4
12-HEPE	8,112 ± 2,893	7,813 ± 2,508	1.0	2,041 ± 533	3,273 ± 1,092	1.6
12-HETE	22,278 ± 2,184	35,561 ± 8,643	1.6	10,836 ± 2,208	14,327 ± 4,034	1.3
17-HDHA	768 ± 119	954 ± 233	1.2	388 ± 49	487 ± 119	1.3
15-HEPE	208 ± 23	209 ± 46	1.0	108 ± 20	123 ± 20	1.1
15-HETE	709 ± 116	898 ± 163	1.3	551 ± 70	570 ± 96	1.0
PD1	n.d	n.d	-	n.d	n.d	-
PDX	n.d	n.d	-	n.d	n.d	-
MaR1	n.d	n.d	-	n.d	n.d	-
RvD1	n.d	n.d	-	n.d	n.d	-
RvD4	21 ± 1.9	24 ± 6.6	1.1	n.d	n.d	-
RvD5	n.d	n.d	-	n.d	n.d	-
**PUFAs**						
AA	2,239,879 ± 231,728	3,618,336 ± 637,031	1.6	2,101,980 ± 151,143	2,985,021 ± 673,892	1.4
EPA	893,062 ± 58,082	1,406,104 ± 232,881	1.6	797,472 ± 86,664	1,192,464 ± 132,302	1.5
DHA	1,103,504 ± 284,606	1,462,360 ± 363,695	1.3	775,753 ± 79,763	985,133 ± 214,450	1.3

#### Effects of Sex and Sex Hormone Depletion on the Expression Levels of Lipid Mediator Biosynthetic Enzymes

**FIGURE 4:**
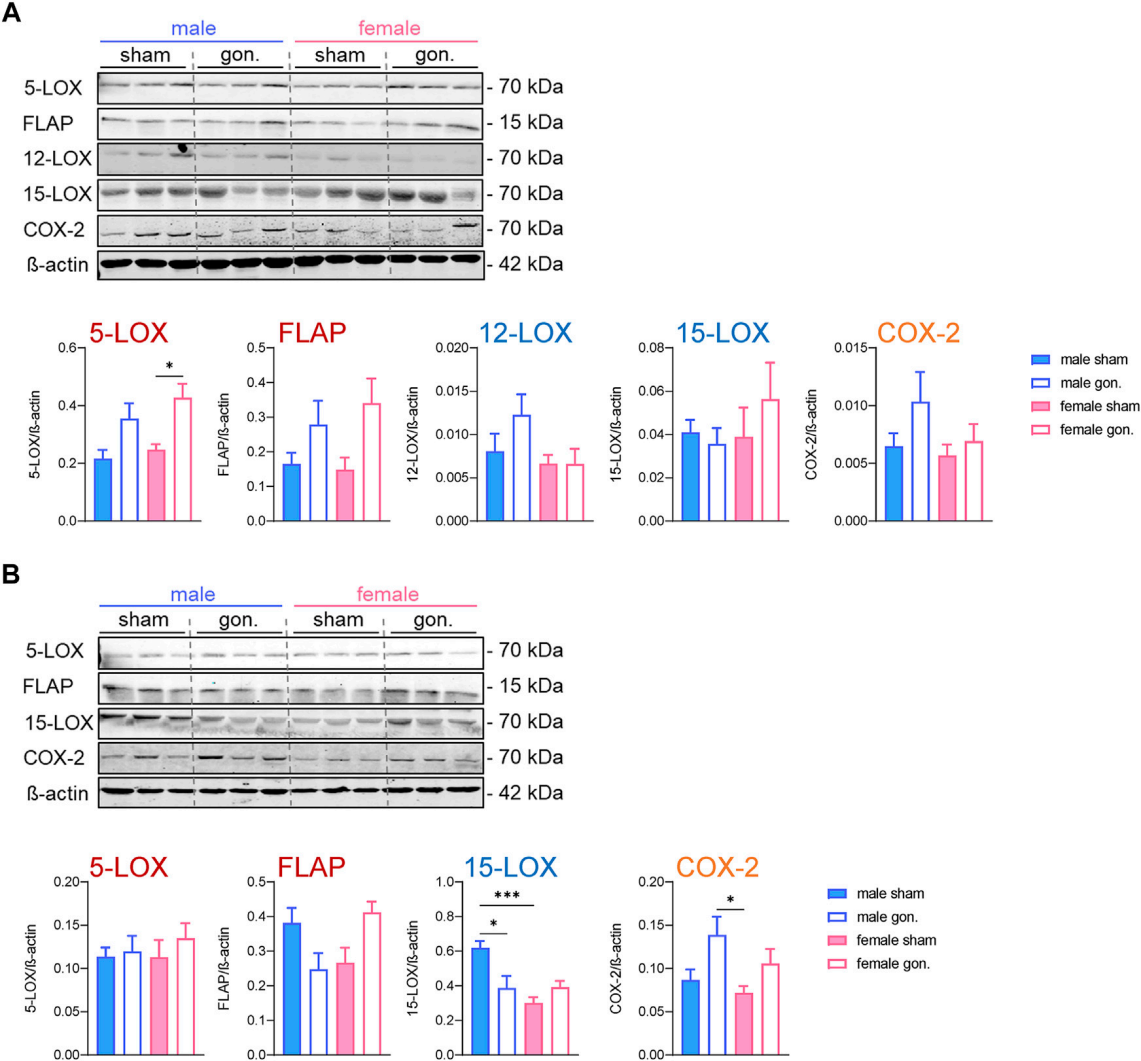
Effect of sex hormone depletion on the expression of LM biosynthetic proteins in peritoneal exudates upon peritonitis induction. Protein levels and the densitometric analysis of 5-LOX, FLAP, platelet-type 12-LOX, 15-LOX, and COX-2 in lysed peritoneal exudates of gonadectomized and non-gonadectomized (sham-operated) male and female mice 4 h **(A)** and 24 h **(B)** after zymosan injection, normalized to *β*-actin. Western blots are shown as representatives from three different mice and given as mean values of n = 4–6; *p < 0.05, **p < 0.01, ***p < 0.001; one-way ANOVA with Tukey’s multiple comparison test for statistical analysis.

The cellular capacity to generate LMs depends primarily on the abundance of the respective biosynthetic enzymes and the availability of free PUFAs as substrates, at least for COX and 12/15-LOX pathways. For 5-LOX, the interaction with FLAP and CLP, the subcellular localization, and phosphorylation at serine residues are additional events that govern product formation (Radmark et al., 2015). Since we already observed some elevated PUFA levels due to gonadectomy, we next studied the amounts of 5-LOX, FLAP, platelet-type 12-LOX, 15-LOX-1, and COX-2 in the peritoneal exudates by Western blot on the protein level. After 4 h of zymosan, no sex differences were evident in the abundance of these proteins in sham-operated animals (Figure 4A). Gonadectomy of male and female mice caused no significant changes in the expression levels of these proteins 4 h post-zymosan, except 5-LOX that was elevated due to ovariectomy in females and some trends toward higher levels of FLAP (Figure 4A). Orchiectomy slightly augmented 5-LOX, FLAP, 12-LOX, and COX-2 proteins in exudates of male mice, which was not significant (Figure 4A). At 24 h post-zymosan, sham-operated male mice displayed significant higher amounts of 15-LOX-1 protein in exudates, which were significantly reduced upon orchiectomy (Figure 4B). For exudate levels of 5-LOX, FLAP, and COX-2 after 24 h, neither significant sex differences nor effects of gonadectomy were apparent (Figure 4B). Together, sex differences in the protein levels of the key LM biosynthetic enzymes/protein are apparent only for 15-LOX-1 in exudates, being superior in male mice after 24 h, while gonadectomy hardly alters the levels of this protein, except the suppression of 15-LOX-1 in males and elevation of 5-LOX in females.

## Discussion

Sex differences in inflammation and in the biosynthesis of LMs have been reported before, and are appreciated as important variables that determine the frequency and severity of inflammation-associated diseases, including many autoimmune diseases (Klein and Flanagan 2016; Pace et al., 2017c; Di Florio et al., 2020; Shabbir et al., 2021). Nevertheless, most studies addressing sex divergences related to LMs focused simply on single selected pathways without covering the entire LM spectrum relevant for progression and resolution of inflammation. Here, we performed the comprehensive LM profile analysis with inflammatory peritoneal exudates and plasma from male and female mice in zymosan-induced peritonitis, a well-established experimental model of acute, self-resolving inflammation (Cash et al., 2009; Rossi et al., 2014), in order to identify potential sex differences in the LM biosynthesis during the inflammatory response. By employing gonadectomy to abrogate endogenous sex hormone production (Cash et al., 2009; Scotland et al., 2011; Rossi et al., 2014), we revealed significant impact of sex hormones on LM formation, particularly in male animals.

Zymosan-induced peritonitis represents an acute local inflammation of the peritoneum with a peak in leukocyte recruitment after 4–12 h (Bannenberg et al., 2005; Rossi et al., 2014) and a subsequent resolution phase after 12–24 h (Schwab et al., 2007). Thus, we have chosen the time point at 4 h as peak and at 24 h as resolution of inflammation in this model. First of all, in healthy mice without zymosan, LM levels were low without apparent sex differences. But clear sex differences in the biosynthesis of pro-inflammatory COX and 5-LOX products were revealed during peritonitis, predominating in males at the peak of inflammation (4 h) and at the onset of resolution (24 h), and a tendency also for 12/15-LOX products that were slightly higher in males after 24 h. This preponderance in males applied to both local LM production at sites of inflammation (exudates) and at least for 5-LOX and 12/15-LOX products, also systemically in the plasma. Intriguingly, at the peak of inflammation 4 h post-zymosan, sex hormone ablation by gonadectomy elevated 12/15-LOX products in the exudates from males, while in females, by contrast, gonadectomy rather decreased free PUFA, 5-LOX, and 12/15-LOX products. In plasma, gonadectomy impaired COX-, 5-LOX–, and 12/15-LOX–derived products when inflammation peaks but not at the resolution phase. Finally, sex differences for the LM biosynthetic enzyme expression were found for 15-LOX-1 dominating in exudates of males at the resolution phase being suppressed by orchiectomy without the striking impact of gonadectomy on other enzymes.

A sex bias in LT formation in zymosan-induced peritonitis was observed before with, however, higher LTB_4_ and cys-LT levels in peritoneal exudates of female mice at least at the very early onset 15–60 min after zymosan injection (Rossi et al., 2014; Pace et al., 2017a), while at later time points (>2 h), a shift to higher LTB_4_ levels in males was obvious (Rossi et al., 2014). The latter observation fits well to the higher 5-LOX product levels in peritoneal exudates and plasma of male mice at 4 h post-zymosan, monitored in the present study. Also, exudates of skin blisters 24 h after cantharidin exposure of male human subjects contained more LTB_4_ than those of female counterparts (Rathod et al., 2017). It thus appears that the sex differences in 5-LOX product formation is temporally regulated during inflammation dominating in females at the early phase but may switch at later stages prevailing in males. Notably, in various *in vitro* studies using short-term (10–30 min) stimulated human neutrophils and monocytes or murine macrophages, cells derived from males produced consistently lower amounts of 5-LOX products vs cells from females (Pergola et al., 2008; Pergola et al., 2011; Pergola et al., 2015; Pace et al., 2017a). It appears that prolonged (>2 h) stimulation of 5-LOX–positive innate immune cells induces regulatory mechanisms in a sex-specific manner (elevation in males and impairment in females) that remain to be explored. Our present data in line with previous results (Pergola et al., 2008; Rossi et al., 2014; Pace and Werz 2020) exclude sex divergences in 5-LOX and the FLAP protein expression at 4 and 24 h in this respect. Rather, distinct temporal subcellular localization of 5-LOX as a highly mobile enzyme (Radmark et al., 2015) is conceivable, which indeed differed in neutrophils, monocytes, and macrophages derived from males vs those from females (Pergola et al., 2008; Rossi et al., 2014; Pace and Werz 2020).

Our current data on sex-specific formation of COX products with higher levels in exudates of male mice at 4 and especially at 24 h post-zymosan fit well to previous studies where PG formation was elevated in male animals during inflammation at 4 h in zymosan-induced mouse peritonitis or at 2 h in carrageenan-induced rat pleurisy (Pace et al., 2017b). In fact, the COX pathway often dominates in males, mainly due to the higher expression of COX-2 (Sullivan et al., 2005), although this may depend on the type of cell, tissue, and organ; on the inflammatory status; and on the sex hormone level related to the age of the subjects (Pace et al., 2017c). Sex differences in PG biosynthesis and modulation by sex hormones were also observed on the cellular level where, for example, neutrophils or platelets from male donors generated more COX products as than those from female counterparts (Mallery et al., 1986; Pinto et al., 1990; Pace et al., 2017b), again supporting our present data with elevated PGs in males. In our study, the protein expression of COX-2, the inducible COX isoform with varying expression during inflammation being causative for massive PG formation (Smith et al., 2011), was not different between sexes, neither at four nor at 24 h post-zymosan. This agrees with the accompanied elevation of pro-inflammatory cytokines that were not significantly different between the sexes.

Formation of 12/15-LOX products including SPM was hardly sex-biased and only elevated in the exudates of male mice 24 h post-zymosan, where accordingly the protein levels of 15-LOX-1 as a key enzyme of SPM formation (Werz et al., 2018; Jordan et al., 2020) dominated significantly in male samples. Along these lines, SPMs were elevated in the male infarcted heart (Pullen et al., 2020). In studies with humans, however, SPM levels tended to be lower in males. Thus, Rathod et al. reported on lower SPM levels in cantharidin-induced skin blisters at the resolution phase of male human subjects (Rathod et al., 2017). Similarly, 24–48 h after myocardial infarction, SPM plasma levels tended to be lower in male patients, which was also affected by the race (Halade et al., 2020). Similarly, plasma levels of 18-HEPE, 14-HDHA, and 17-HDHA were lower in male metabolic syndrome patients, although SPM levels were not different (Barden et al., 2015). Of interest, not only SPMs (i.e., RvD5) but also PGE_2_ were lower in the lungs of male mice after exposure to ozone along with reduced AA and DHA levels compared with females, but ovariectomy had no impact on eicosanoid and SPM production despite depleted pulmonary DHA concentrations (Yaeger et al., 2021). Conversely, in healthy volunteers treated with dexamethasone, the plasma levels of 17-HDHA, RvD1, AT-RvD1, and RvE2 were higher in males (Barden et al., 2018). Also, higher SPM levels were found in emotional tears from men than in those derived from females (English et al., 2017), while retinal RvD1 levels in aged mice were again lower in males vs females (Trotta et al., 2021). Therefore, sex differences in SPM formation are obvious but inconsistent without clear preponderance in one gender, depending on experimental settings, species, race, age, and additional factors that may impact SPM formation.

To gain further insights into how sex impacts LM biosynthetic pathways during acute inflammation, we studied how ablation of sex hormones would modulate LM formation using gonadectomy. Several studies revealed striking suppression of LT formation by androgens in activated human neutrophils (Pergola et al., 2008; Pace et al., 2017a) and monocytes (Pergola et al., 2011), and in murine peritoneal macrophages (Rossi et al., 2014; Pace et al., 2017a). In a mouse asthma model, testosterone treatment during the sensitization phase lowered LT levels in the lung of female animals (Cerqua et al., 2020). Conversely, the ablation of testosterone in male mice by orchiectomy increased LTB_4_ and cys-LT formation in the peritoneum 15–30 min post-zymosan i.p. injection (Rossi et al., 2014). Besides androgens, also progesterone was found to rapidly suppress the LT biosynthesis, especially in human monocytes (Pergola et al., 2015), while estradiol increased the 5-LOX activity in isolated kidneys of metabolic syndrome female rats (Zuniga-Munoz et al., 2015). In our present study, decreased 5-LOX products in peritoneal exudates and plasma of orchiectomized male mice were evident at 4 h post-zymosan; however, at 24 h, 5-LOX products were rather elevated in orchiectomized animals. Despite decreased 5-LOX product formation at 4 h, gonadectomy elevated the protein levels of 5-LOX and FLAP in both sexes. These data suggest that modulation of the 5-LOX pathway by sex hormones proceeds by other processes than the regulation of 5-LOX and the FLAP protein expression, such as the 5-LOX/FLAP interaction, and seemingly depends also on the time point or phase of the inflammatory process (Pergola et al., 2008; Pace et al., 2017a).

We found that sex hormone depletion had only moderate and rather inconsistent impact on PG formation in the zymosan-challenged peritoneum along with slight elevations of COX-2 protein. However, in the plasma of both sexes, the elevated PG levels at the peak of inflammation (4 h) were strongly repressed by gonadectomy, implying that sex hormones are required for systemic induction of COX pathways. Others found that in the brain, estrogen, and/or progesterone suppress the COX-2 pathway, as ovariectomy augmented the COX-2 expression and PGE_2_ in female rat hypothalamus (Brito et al., 2016), while estrogen and progesterone treatment of ovariectomized rats suppressed the LPS-induced COX-2 expression (Mouihate and Pittman 2003). In the kidney, however, the mPGES-1 expression was elevated after orchiectomy without changes of COX and cPGES (Sullivan et al., 2005). Together, sex hormones influence COX product formation with not only up-regulatory effects but also down-regulatory effects, which may depend on the organ/tissue and the phase of the inflammatory process.

Little is known about the impact of sex hormones on the 12/15-LOX pathway and related SPM formation. In the present study, the most pronounced effects of gonadectomy were indeed obvious for the 12/15-LOX pathway, where orchiectomy strongly increased 12/15-LOX product formation without the elevation of 15-LOX-1 protein in the peritoneum, but not in the plasma after 4 h; in contrast, ovariectomy was rather suppressive. At 24 h, gonadectomy elevated 12/15-LOX product levels in the peritoneum and plasma of both sexes, with more pronounced effects in males. Thus, androgens may have suppressive and estrogen/progesterone stimulatory impact on 12/15-LOX at the site of inflammation. This fits well to results by others showing that application of estradiol increases the 12-LOX and 15-LOX-2 expression and activity in human umbilical vascular smooth muscle cells (Somjen et al., 2016), and enhances the 12-LOX activity in rat platelets *in vivo* (Chang et al., 1982). Moreover, chronic hyperandrogenism decreased the protein expression of 12-LOX in prepuberal mice (Elia et al., 2011), supporting our hypothesis of a detrimental impact of androgens on the 12/15-LOX pathway and thus on SPM formation.

## Conclusion

Using UPLC–MS-MS–based profiling of a broad spectrum of LMs in the peritoneum and plasma of male and female mice during the course of acute inflammation revealed significant sex differences for defined LM biosynthetic pathways at the inflamed site. Thus, in peritoneal exudates, pro-inflammatory COX and 5-LOX products predominate in males vs females at the peak of inflammation (4 h) and at the onset of resolution (24 h), while in plasma, only 5-LOX products dominated in males with minor magnitude. Neither the availability of PUFAs as LM substrates nor the expression of the, respective, key enzymes COX-2 and 5-LOX/FLAP in the exudates differed between males and females, and therefore do not readily account for these sex divergences. However, the slightly higher 12/15-LOX products in male exudates after 24 h correlate to elevated 15-LOX-1 protein levels. Our results with gonadectomized mice, and thus impaired sex hormone levels, indicate a pronounced suppression of 12/15-LOX product formation in males by androgens without such impact of estrogen and progesterone, again without clear correlation to PUFA supply and the 15-LOX-1 expression. Moreover, sex hormones influence COX and 5-LOX product formation with not only up-regulatory effects but also down-regulatory effects, which may depend on the organ/tissue and the phase of the inflammatory process. In summary, our data add to the obvious sex differences in LM biosynthetic networks that are, however, inconsistent in view of the respective pathway and the phase of the inflammatory process, and are seemingly further governed by the species, the race, the age of studied subjects, and the experimental settings. Consequent consideration of the sex in experimental and clinical studies of LM biosynthesis and biology may help to better understand how this complex sex bias is regulated, especially by sex hormones.

## Data Availability

The raw data supporting the conclusion of this article will be made available by the authors, without undue reservation.

## Abbreviations

AA, arachidonic acid; COX, cyclooxygenase; cPLA2, cytosolic phospholipase A2; DHA, docosahexaenoic acid; EPA, eicosapentaenoic acid; FLAP, 5-lipoxygenase-activating protein; LPS, lipopolysaccharide; HDHA, hydroxy-DHA; LM, lipid mediator; LOX, lipoxygenase; LT, leukotriene; LX, lipoxin; NSAID, nonsteroidal anti-inflammatory drugs; PUFAs, polyunsaturated fatty acids; PG, prostaglandin; Rv, resolvin; SPM, specialized pro-resolving mediators; TX, thromboxane.
